# Clinical features and outcome of 10 dogs with suspected idiopathic vestibular epilepsy

**DOI:** 10.1111/jvim.17046

**Published:** 2024-03-21

**Authors:** Tania Al Kafaji, Fabio Tocco, Samuel Okonji, Antonella Gallucci

**Affiliations:** ^1^ Veterinary Neurological Center “La Fenice” Selargius Italy; ^2^ Department of Veterinary Medical Sciences University of Bologna Bologna Emilia‐Romagna Italy

**Keywords:** canine, electroencephalography, paroxysmia, seizures, vertigo

## Abstract

**Background:**

In humans, vestibular epilepsy (VE) is described as focal seizures with transient signs of vestibular disease. In dogs, 2 cases of vestibular episodes, called vestibular paroxysmia, are reported.

**Hypothesis/objectives:**

The objective of this study was to define the clinical features, phenotypical manifestation, and outcome of suspected VE in dogs.

**Animals:**

Ten dogs with recurrent vestibular episodes.

**Methods:**

Retrospective study. Medical records between 2009 and 2023 were reviewed, and dogs with a normal neurological examination, a history of transient signs of vestibular disease, absence of abnormalities detected on blood exams and brain magnetic resonance imaging (MRI) or computed tomography (CT), besides a minimum 10‐month follow‐up were included. Clinical improvement was defined as a ≥50% reduction in frequency or the cessation of clinical signs after the onset of antiseizure medications (ASMs).

**Results:**

Pugs were the most prevalent breed (5/10; 50%). In 2 cases, additional generalized tonic‐clonic (GTC) seizures were reported. MRI exam was performed in most cases (9/10; 90%), whereas 1 dog underwent a CT scan (1/10; 10%). Electroencephalography (EEG) was carried out in 3 dogs that showed interictal spikes in the fronto‐temporal and fronto‐parietal areas. All cases received ASMs, with clinical improvement in 10/10 dogs (100%).

**Conclusion and clinical importance:**

The presence of GTC seizures, EEG interictal spikes, and responsiveness to ASMs supported the hypothesis of an epileptic origin of vestibular episodes and thus the existence of VE in these dogs, with a presumed idiopathic cause and apparent favorable outcome.

**Abbreviations**
AED: antiepileptic drugs
B: potassium bromide
CNS: central nervous system
CSF: cerebrospinal fluid
CT: computed tomography
EEG: electroencephalography
FLAIR: fluid‐attenuated inversion recovery
GTC: generalized tonic‐clonic
LEV: levetiracetam
MRI: magnetic resonance imaging
PB: phenobarbital
PCR: polymerase chain reaction
PSOM: primary secretory otitis media interna
T1W: T1‐weighted
T2W: T2‐weighted
TIA: transient ischemic attack
TNCC: total nucleated cell count
TPO: temporo‐parietal‐occipital
VE: vestibular epilepsy
VP: vestibular paroxysmia

## 1 INTRODUCTION

Vestibular epilepsy (VE) is described in human medicine as focal seizures with signs of vestibular disease as the only or main symptoms.^1^ Clinical signs can range from mild disequilibrium to dizziness and vertigo.^2^, ^3^, ^4^ Vestibular seizures are characterized by a short duration, of a few seconds, and an abrupt ending, although longer episodes lasting several minutes are also described.^4^

In human literature, many studies have focused on this kind of epilepsy. The human “vestibular cortex” is defined as a complex neuronal network involved in postural functions and eye movements.^5^ Its anatomical localization has been investigated using cortical electrical stimulation, electroencephalography (EEG), and functional magnetic resonance imaging (MRI) studies.^2^, ^6^, ^7^ The temporal and parietal lobes are the predominant areas involved,^4^, ^5^, ^7^ followed by, at a lower frequency, the occipital and frontal cortexes.^2^ Specifically, a temporo‐peri‐Sylvian vestibular cortex was identified through intracranial electrical stimulation.^4^, ^7^

Brain MRI studies performed in human patients with vestibular seizures have identified no brain abnormalities^3^ or brain structural disorders such as congenital malformations or ischemic lesions.^5^ EEG studies have shown interictal spikes and sharp waves at the level of the parietal and temporo‐parietal‐occipital junction areas.^3^ The responsiveness to antiepileptic treatment is well described and present in up to 90% of cases.^2^, ^3^, ^4^

An important differential diagnosis of VE is vestibular paroxysmia (VP). It is defined as episodes of spinning or non‐spinning vertigo, lasting from seconds to minutes, which occur spontaneously or after changes in body position in the absence of other potential underlying differential diagnoses.^8^

In veterinary medicine, VP has been described in 2 dogs: 1 was correlated with a suspected transient ischemic attack (TIA) because of the presence of systemic hypertension^9^ and the other with a suspected atypical form of Ménière's disease.^10^

It can be highly complex and challenging to discriminate between VP and VE. In human medicine, a diagnosis of VE is strongly supported by abnormal electroencephalography (EEG) findings compatible with seizures activity and responsiveness to antiepileptic treatment.^4^

Vestibular syndrome associated with peripheral or central vestibular dysfunction, caused by structural, metabolic, or idiopathic conditions is largely reported in veterinary medicine. The clinical signs do not usually present as transient occurrence of fits and depending on the etiology, they can get worse, improve, or remain stable.^11^, ^12^, ^13^, ^14^ Differently, in this study cohort, the signs of vestibular disease were characterized by recurrent episodes, with return to a normal state among them, as happens in epileptic seizures. Similar recurrent episodes of vestibular disease of suspected epileptic origin are not described in dogs. This study attempts to define the clinical features, phenotypical manifestations, and outcome of dogs with suspected VE.

## 2 MATERIALS AND METHODS

A retrospective study was conducted to search for the medical records of dogs referred to the Veterinary Neurological Center “La Fenice” (Cagliari, Italy) and the Department of Veterinary Medical Sciences of University of Bologna (Bologna, Italy) between January 2009 and May 2023 for suspected vestibular seizures or VP. Inclusion criteria were (1) the presence of complete medical records comprehensive of signalment, history, neurological examination and diagnostic findings; (2) a normal neurological examination performed by a board‐certified veterinary neurologist (AG) or a veterinary neurology resident‐in‐training (TA); (3) considering the definition of epilepsy reported by the task force consensus,^15^ a history of at least 2 episodes >24 h apart characterized by the sudden onset and abrupt ending of clinical signs of vestibular disease (such as a loss of balance, drifting, rolling, and nystagmus) lasting from a few seconds to minutes; (4) at least 1 episode documented by video recording; (5) the exclusion of reactive seizures by the evaluation of normal laboratory results at the presentation of vestibular episodes, including the total cell blood count, the serum biochemistry profile (alanine aminotransferase, aspartate aminotransferase, alkaline phosphatase, urea, creatinine, total protein, albumin, total bilirubin, glucose, cholesterol, triglycerides, sodium, potassium, chloride, calcium, and phosphate), and fasting bile acids and/or ammonia; (6) the absence of brain MRI or computed tomography (CT) exam abnormalities; (7) a minimum follow‐up of 10 months from the time of presentation. Further clinical characterization of the different vestibular episodes was obtained by the owner's description, through video footage, or during clinical examination. Dogs which experienced additional generalized tonic‐clonic (GTC) seizures were also included in the study.

Data collected from each dog include signalment, anamnesis, drugs administered before presentation, diagnostic investigations performed, medical treatment, results of brain MRI or CT exam, and clinical outcome. The results of cerebrospinal fluid (CSF) analysis and/or EEG were included when present. The MRI exam was performed with a 0.3 Tesla scanner (Hitachi Airis II) or with a 0.22 Tesla (Paramed Mr J 2200). T2‐weighted (T2W) images were acquired in the sagittal and transverse planes. Fluid‐attenuated inversion recovery (FLAIR) was obtained in the dorsal plane. T1‐weighted (T1W) images were acquired in the sagittal, dorsal, and transverse planes before and after intravenous gadoteric acid 0.1 mmol/kg injection. The CT images (Somatom Spirit, Siemens Healthcare, Germany) were obtained before and after intravenous (600 mgI/kg) bolus injections of nonionic, low‐osmolar iodinated contrast agent Iopamidol. MRI and CT were evaluated by a board‐certified veterinary neurologist (AG). A total nucleated cell count of CSF samples between 0 and 5 cells/μL was identified as normal. Protein concentrations of <30 mg/dL for CSF sampled from the cisterna magna and <45 mg/dL for lumbar puncture were considered normal.^16^

Follow‐up information was obtained by medical records, telephone interviews, or clinical re‐evaluation. Clinical improvement was defined as a reduction in frequency or the cessation of clinical signs after starting medical treatment. A reduction of ≥50% in vestibular episodes has been adopted as the definition of ASMs efficacy in most of the studies of epilepsy in dogs.^17^, ^18^, ^19^, ^20^, ^21^, ^22^, ^23^ The persistence of signs of vestibular disease with <50% reduction or increased frequency despite therapeutic intervention was classified as a failure to respond to treatment. Data regarding euthanasia or death, whether correlated or not to the neurological disorder, were noted.

## 3 RESULTS

### 3.1 Animals

Ten dogs met the inclusion criteria and were enrolled in the study. Pugs were the most prevalent breed (5/10, 50%). There were 6 male dogs (6/10, 60%) and 4 female dogs (4/10, 40%; Table 1). At the time of presentation, the dogs' age ranged from 8 months to 13 years (media, 7 years).

**TABLE 1:** Signalment of the population study.

Dogs	10
Breeds	Pugs (*n* = 5; 50%)
	Mixed breed (*n* = 3; 30%)
	Staffordshire Bull Terrier (*n* = 1; 10%)
	Cavalier King Charles Spaniel (*n* = 1; 10%)
Age	m = 80 months (range 8–156)
Sex	Male *n* = 6 (60%)
	Male Neutered *n* = 0 (0%)
	Female *n* = 3 (30%)
	Female Spayed *n* = 1 (10%)

Abbreviations: m, media; *n*, number of dogs.

### 3.2 Clinical features

The clinical signs included the acute onset of vestibular ataxia with lateral drifting, rolling, head tilt, and horizontal nystagmus (Videos 1 and 2). Signs of vestibular disease lasted from <1 min to a maximum duration of 1 hour (Table 2). They were characterized by an abrupt ending followed by an immediate return to normality in the absence of other post ictal signs such as disorientation or compulsive behavior. During the episodes, the dogs seemed responsive to the owner's voice. Episodes occurred with variable frequency, ranging from daily to a few per year (Table 2).

**VIDEO 1:** Video recording of a pug dog with acute onset of transient vestibular signs characterized by head tilt and drifting to the right side, vertical and horizontal nystagmus with the fast phase directed to the right side. Embedded Video 1 placeholder: image taken at 00:00:36 s.

**VIDEO 2:** Video recording of a pug dog with acute onset of loss of balance and horizontal nystagmus with the fast phase directed to the left side. Embedded Video 2 placeholder: image taken at 00:00:05 s.

**TABLE 2:** Characterization of vestibular episodes in the population study.

Dogs	10 (100%)
Clinical signs	
Vestibular drifting	9/10 (90%)
Nystagmus	8/10 (80%)
Rolling	2/10 (20%)
Head tilt	3/10 (30%)
Circling	1/10 (10%)
Duration	
<1 min	1/10 (10%)
1–5 min	2/10 (20%)
5–30 min	2/10 (20%)
30‐60 min	5/10 (50%)
Frequency	
Daily (≥1/day)	1/10 (10%)
≥1/week	2/10 (20%)
≥1/month	5/10 (50%)
3 months interval	1/10 (10%)
≥3 months interval	1/10 (10%)

### 3.3 Investigations and diagnosis

A normal brain MRI exam was obtained in 9 dogs (9/10, 90%), whereas a CT scan was done in a dog and revealed no abnormalities (1/10, 10%). CSF samples were collected from 4 dogs (4/10, 40%) and were within the normal range.

Two dogs (2/10, 20%) had isolated GTC seizures in addition to signs of vestibular disease. In 1 case, there was a history of 2 years of GCT seizures, occurring approximately every 6 months, before the appearance of signs of vestibular disease. In such dog, brain MRI and CSF analysis were normal, and an idiopathic epilepsy was suspected. Another dog was referred for recurrent signs of vestibular disorder; the brain MRI was normal, and the vestibular episodes disappeared after starting ASMs. GTC seizures occurred 3 years later, a few months after the discontinuation of therapy. An EEG exam was performed in 3 dogs using a 16‐channels system with a reference montage. The dogs were sedated with dexmedetomidine 1 mcg/kg intravenously (IV). Anesthesia was obtained by administration of a single bolus of propofol 1 mg/kg IV followed by a constant rate infusion (CRI) at dosage ranging from 0,1‐0,2 mg/kg/min. In 1 dog, affected by GTC seizures in addition to episodes characterized by drifting and rolling on the right side, occasional interictal spikes were observed in the right fronto‐temporal region. The other cases that underwent the EEG exam were affected only by episodes of vestibular disease without GTC seizures. In both, interictal spikes and slow‐waves were detected in the left frontal and fronto‐parietal areas. In 1 of these dogs, a left lateralization of signs of vestibular disease had been noted.

### 3.4 Therapeutic interventions and outcome

Due to the increased frequency or severity of episodes, ASMs were administered in all cases (10/10, 100%). All dogs except 1 received levetiracetam (LEV; Keppra, GlaxoSmithKline, Jedda, Kingdom of Saudi Arabia or Matever, Pharmathen, Attica, Greece; 9/10, 90%; at dosages ranging from 20 to 34 mg/kg q8h), in monotherapy (5/10, 50%) or in combination with phenobarbital (PB; Soliphen, Dechra Veterinary Products, Torino, Italy or Gardenale, Sanofi S.r.l., Milano, Italy; 3/10, 30%; at dosages ranging from 2.5 to 3.2 mg/kg every 12 h) or Gabapentin (GBP; Teva Italia S.r.l., Milano, Italy; 1/10, 10%, at dosage of 10 mg/kg every 8 h). PB was administered as monotherapy only in 1 case (1/10, 10%; at a dosage of 3.2 mg/kg every 12 h; Table 3).

**TABLE 3:** Therapeutic interventions in the population study.

Drugs	*n* = 10 (100%)	Drugs dose (m) (range)
LEV	5/10 (50%)	22,5 mg/kg (20–30) Q8h
LEV + PB	3/10 (30%)	LEV 29,6 mg/kg Q8h (25–34); PB 3,1 Q12h (2,7‐3.5)
LEV + GBP	1/10 (10%)	LEV 20 mg/kg Q8h; GBP 10 mg/kg Q8h
PB	1/10 (10%)	3.2 mg/kg Q12h

Abbreviations: B, potassium bromide; GBP, gabapentin; LEV, levetiracetam; *n*, number of dogs; m, media; PB, phenobarbital.

The follow‐up for each dog ranged from a minimum of 10 months to a maximum of 4 years from the time of presentation (median, 961 days; range, 272–2169 days). Clinical improvement with a decrease in the frequency, duration, and severity of vestibular episodes was observed in all cases (10/10, 100%). In more detail, in 5 dogs (5/10, 50%), vestibular episodes disappeared after the initiation of treatment with LEV in monotherapy. A reduction in the frequency or severity of signs of vestibular disorder was observed in 5 cases (5/10, 50%). One of these dogs received PB only and the frequency of episodes reduces from daily to once every 2 months. In another case, LEV was added to GBP after 1 year from the initiation of therapy, because of reoccurrence of a single vestibular episode. The other dogs were in combination therapy with LEV and PB. Of these, 2 dogs were initially treated with LEV monotherapy without the improvement of vestibular episodes. In 1 case, PB was added after a few months due to the absence of improvement with LEV and signs of vestibular disease disappeared for 6 months until the dog dead for unrelated reasons. In another dog, vestibular episodes stopped after the beginning of therapy with LEV, but PB was added 3 years later due to the recurrence of vestibular episodes and the onset of cluster GCT seizures secondary to LEV therapy discontinuation (owner's decision; Table 3). During the following 6 months of therapy with PB this dog did not experience further episodes.

At the time of writing, 2 dogs (2/10, 20%) were dead due to causes other than the neurological disease (aging).

## 4 DISCUSSION

This study describes the phenotypic clinical features and the outcome of suspected idiopathic vestibular seizures in dogs. Veterinary literature regarding data focused on this topic is lacking. In all cases included in the study, the reported vestibular episodes could be consistent with suspected VE or VP. Although clinical differentiation among these disorders is challenging, the presence of additional GTC seizures, interictal EEG spikes at the level of the fronto‐parietal and fronto‐temporal areas, and positive responsiveness to antiepileptic therapy supported the hypothesis of an epileptic origin of vestibular episodes in the cohort of this study. Therefore, we can assume the possible existence of VE in dogs, with an idiopathic cause and with a wide age range at the presentation of clinical signs.

The prevalent breed in our study was Pug dogs. Although such results must be interpreted carefully due to the small number of cases, we can speculate that as a higher seizure prevalence was reported in Pug dogs,^24^ maybe they can be predisposed to VE. However, in the authors opinion, further studies with larger cohort are needed to investigate this aspect.

In human medicine, the underlying mechanism of VP is thought to be nerve‐vessel contact, with the major involvement of the anterior inferior cerebellar artery. The vessel pulsatile compression exerted on the 8th cranial nerve leads to a focal mechanical nerve injury with associated demyelination and subsequent development of nerve hyperexcitability.^25^ The diagnosis of VP is mainly based on clinical features, including a high frequency, at least 10 per day, a short duration of attacks (<60 s), and responsiveness to treatment with carbamazepine and oxacarbazepine, that primarily block the use‐dependent fast voltage gated sodium channels.^25^ High‐resolution brain MRI using specific sequences could be helpful in identifying a neurovascular abnormality, with a reported sensitivity of 100% but a specificity of about 65%.^26^, ^27^ In none of the cases in this study, diagnostic imaging detected a compression of the vestibulocochlear nerve. However, the use of a low‐field MRI scan could potentially have failed to identify it. Unlike human VP, in our cohort, the duration of vestibular attacks lasted from several minutes up to an hour. Regarding the treatment of VP, the first‐line drugs used in human medicine are carbamazepine and oxcarbazepine and responsiveness to this drugs is considered part of the diagnostic criteria for VP.^28^ Conversely, in our study sample, no dog was treated with a sodium channel blocker. In addition, a high remission rate is observed in human medicine with discontinuation of medical therapy.^28^ Conversely, reduction or ending of vestibular attacks without treatment with ASMs was not observed in any case. In the veterinary literature, presumed VP was described in 2 cases. In 1 case, episodes of vestibular disease occurred in a 9‐year‐old dog with absence of abnormalities on MRI and CSF analysis, in which primary hypertension was detected and TIAs were considered the final diagnosis. In contrast to our study, in this dog, signs of vestibular disease resolved with the sole utilization of antihypertensive drugs, without ASMs.^9^ In addition, blood pressure was tested in all dogs of our study, as part of the standard monitoring under anesthesia, and hypertension was not detected in any case. In the other case, VP was described in a 4‐month‐old dog with normal CT, CSF, and brainstem auditory evoked responses (BAER) test, in which antiepileptic treatment with LEV, FB, and imepitoin failed in improve clinical signs and VP occurred on a daily basis.^10^

In human literature, the term “epileptic vertigo or dizziness” is used to describe signs of vestibular disease triggered by focal epileptic discharges.^2^ VE has been reported occurring with a low incidence (9,3%) in people.^2^ Clinical signs range from mild disequilibrium to rotational vertigo. Additional clinical features are sensational, motor, autonomic, and emotional manifestations.^2^ The presence of nystagmus associated with VE occurred with a low frequency in human patients. It has been speculated that the simultaneous activation of different cortical areas responsible for eye movements and vertigo/dizziness during VE is uncommon.^2^ Conversely, in this study vestibular nystagmus was observed in most dogs. Moreover, in human medicine, “isolated VE”, defined as symptoms of vestibular disease not accompanied by additional epileptic seizures signs, occurred in a lower proportion than “non‐isolated VE.”^2^ It has been suggested how bursts of epileptic activity characterized by a short duration could led to isolated VE. Conversely, the onset of additional seizures could be the consequence of a longer spontaneous epileptic activity.^2^ In our cohort, GTC seizures were observed only in 2 dogs. Basing on our results, we could speculate that in veterinary medicine isolated VE occurred more frequently, but further studies with larger samples are needed to confirm this assumption. In 1 of these dogs, GTC seizures started later, after the discontinuation of antiepileptic treatment used to treat VP/VE. In this dog, EEG exam detected interictal spikes in the fronto‐temporal region, confirming the presence of seizure activity. Interestingly, also in people with VE, ictal/interictal EEG studies showed the presence of seizure activity predominantly at the level of the temporal lobe.^2^ In the other 2 cases with only signs of vestibular disorder, interictal spikes and slow‐waves were detected at the level of the left fronto‐parietal and frontal areas. In humans, although with a lower frequency, the parietal, occipital, and frontal areas might also be involved.^2^, ^3^, ^5^ Specifically, a lateral cortical temporo‐parietal area, known as the “temporo‐peri‐Sylvian vestibular cortex” has been identified through cortical electrostimulation.^7^ It comprises the superior aspect of the temporal area and the lateral part of the inferior parietal lobe.^7^ These sites are specifically involved in receiving and processing vestibular signals and give rise to the afferent fibers directed to the vestibular nuclei. In addition, it has been demonstrated how the activation of distinct areas of this vestibular cortex could lead to different clinical signs.^4^, ^7^ Specifically, 3 anatomical sites responsible for coding spatial information in a 3‐dimensional way were detected and include the parietal operculum, the temporal neocortex and the mesial parietal cortex.^4^, ^7^ The identification of EEG interictal spikes in the temporal, parietal, and frontal lobes in 3 dogs could potentially support our hypothesis of an epileptic origin underlying signs of vestibular disease. In 2 cases the side of the brain involved in the EEG changes was the same as neurological signs reported as rolling, drifting and head tilt. The correlation between EEG activity and clinical signs could be another relevant finding suggesting a potential epileptic origin of the episodes. In human medicine, the role of ictal motor signs lateralization in predicting the epileptogenic foci has been investigated. Head turn was predominantly associated with contralateral electrostimulation of frontal eye field and superior collicus due to the control activity on spinal cord motoneurons and potential activation of the sternocleidomastoid muscle ipsilateral to the hemisphere of seizures onset.^29^ An ipsilateral head deviation has also been reported occurring at the end of TCG seizures. It has been related to the activation of 1 hemisphere after the exhaustion or inhibition of the other.^30^ Moreover, ipsilateral head movements and ipsilateral automatisms were observed in human patients with temporal lobe epilepsy.^30^, ^31^ In fact, various mechanisms correlated to different cortical generator centers are thought to be involved in ictal head movements.^32^ In veterinary medicine, vestibular conscious perception pathways originating from vestibular nuclei, passing through the controlateral medial geniculate nuclei and terminating into cerebral cortex are described.^33^ In veterinary medicine, the role of cortical electrostimulation on lateralization of signs of vestibular disease has not been investigated.

In the other dog with GTC seizures, vestibular episodes occurred years after the onset of epileptic seizures. It has been suggested how recurrent seizures in people affected by idiopathic epilepsy could lead to functional damage to the vestibular cortex with the later onset of VE.^1^

Up to 90% of human patients with VE have a favorable response to ASMs. This aspect was also observed in this study. Although responsiveness to ASMs could support an epileptic origin of vestibular episodes, caution must be taken when trying to use it as a discriminatory criterion.^2^

EEG exam was not performed in all dogs, which could be a major limitation of the study. EEG analysis is considered an important diagnostic tool to differentiate suspected seizures from paroxysmal events. Although the presence of seizure activity was detected in all dogs receiving an EEG exam, further prospective studies with a complete diagnostic plan, including EEG exam, are needed to evaluate this aspect.

CSF collection was performed in 4 dogs, and this could have led to failure in detecting disease conditions even in the presence of a normal brain MRI study. However, progression of clinical signs was not observed in any of the cases without CSF analysis which makes unlikely the presence of inflammatory/infectious or neoplastic diseases.

The inclusion of the dog with a CT scan could also potentially have affected the final diagnosis in this case. In fact, MRI exam is considered the preferred diagnostic method for investigation of brain disease, allowing a better soft tissues resolution.^34^ Nevertheless, in human medicine CT is the advanced diagnostic modality used in the initial evaluation of patients with suspected brain lesions. Also in veterinary medicine, a variety of CT protocols have been developed in the last years to detect brain abnormalities.^35^

Although the small number of cases included in this study represents a limitation, these collected data could represent a preliminary step in the attempt to describe potential idiopathic VE in dogs. The small number of cases included over a long period of time, could suggest how this form of suspected epilepsy occurs with a low incidence in dogs as also reported in human medicine.^3^

## CONFLICT OF INTEREST STATEMENT

Authors declare no conflict of interest.

## OFF‐LABEL ANTIMICROBIAL DECLARATION

Authors declare no off‐label use of antimicrobials.

## INSTITUTIONAL ANIMAL CARE AND USE COMMITTEE (IACUC) OR OTHER APPROVAL DECLARATION

Authors declare no IACUC or other approval was needed.

## HUMAN ETHICS APPROVAL DECLARATION

Authors declare human ethics approval was not needed for this study.

## References

[1] Hamed SA , Tohamy AM , Oseilly AM . Vestibular function in adults with epilepsy of unknown etiology. Otol Neurotol. 2017;38(8):1217‐1224. 28742631 10.1097/MAO.0000000000001513

[2] Tarnutzer AA , Lee SH , Robinson KA , Kaplan PW , Newman‐Toker DE . Clinical and electrographic findings in epileptic vertigo and dizziness: a systematic review. Neurology. 2015;84(15):1595‐1604. 25795644 10.1212/WNL.0000000000001474 PMC4408281

[3] Hewett R , Guye M , Gavaret M , Bartolomei F . Benign temporo‐parieto‐occipital junction epilepsy with vestibular disturbance: an underrecognized form of epilepsy? Epilepsy Behav. 2011;21(4):412‐416. 21704564 10.1016/j.yebeh.2011.05.017

[4] Hewett R , Bartolomei F . Epilepsy and the cortical vestibular system: tales of dizziness and recent concepts. Front Integr Neurosci. 2013;7:73. 24273498 10.3389/fnint.2013.00073 PMC3822407

[5] Morano A , Carnì M , Casciato S , et al. Ictal EEG/fMRI study of vertiginous seizures. Epilepsy Behav. 2017;68:51‐56. 28109990 10.1016/j.yebeh.2016.12.031

[6] Andrade‐Machado R , Benjumea‐Cuartas V . Temporal plus epilepsy: Anatomo‐electroclinical subtypes. Iran J Neurol. 2016;15(3):153‐163. 27648177 PMC5027151

[7] Kahane P , Hoffmann D , Minotti L , Berthoz A . Reappraisal of the human vestibular cortex by cortical electrical stimulation study. Ann Neurol. 2003;54(5):615‐624. 14595651 10.1002/ana.10726

[8] Karamitros A , Kalamatianos T , Stranjalis G , Anagnostou E . Vestibular paroxysmia: clinical features and imaging findings; a literature review. J Neuroradiol. 2022;49(2):225‐233. 34364914 10.1016/j.neurad.2021.07.007

[9] Bentley RT , March PA . Recurrent vestibular paroxysms associated with systemic hypertension in a dog. J Am Vet Med Assoc. 2011;239(5):652‐655. 21879966 10.2460/javma.239.5.652

[10] Liatis T , Balla S , Baka R , et al. Recurrent peripheral vestibular paroxysm in a young dog: it is a new entity? Proceedings of the 31st ESVN‐ECVN Symposium. Copenhagen: Denmark; 2018.

[11] Orlandi R , Gutierrez‐Quintana R , Carletti B , et al. Clinical signs, MRI findings and outcome in dogs with peripheral vestibular disease: a retrospective study. BMC Vet Res. 2020;16(1):1‐10. 32450859 10.1186/s12917-020-02366-8 PMC7249679

[12] Lowrie M , Vetmb MA . Vestibular disease: diseases causing vestibular signs. Compend Contin Educ Vet. 2012;34(7):E2. 22847321

[13] Negrin A , Cherubini GB , Lamb C , Benigni L , Adams V , Platt S . Clinical signs, magnetic resonance imaging findings and outcome in 77 cats with vestibular disease: a retrospective study. J Feline Med Surg. 2010;12(4):291‐299. 19932040 10.1016/j.jfms.2009.10.001 PMC11135580

[14] Rossmeisl JH . Vestibular disease in dogs and cats. Vet Clin Small Animal Pract. 2010;40(1):81‐100. 10.1016/j.cvsm.2009.09.007 19942058

[15] Berendt M , Farquhar RG , Mandigers PJ , et al. International veterinary epilepsy task force consensus report on epilepsy definition, classification and terminology in companion animals. BMC Vet Res. 2015;11(1):1‐11. 26316133 10.1186/s12917-015-0461-2 PMC4552272

[16] Levine GJ , Cook JR . Cerebrospinal fluid and central nervous system cytology. In: Valenciano AC , ed. Cowell RL: Cowell and Tyler's Diagnostic Cytology and Hematology of the Dog and Cat. 5th ed. St. Louis (MO): Elsevier; 2020:210‐228.

[17] Volk HA , Matiasek LA , Luján Feliu‐Pascual A , Platt SR , Chandler KE . The efficacy and tolerability of levetiracetam in pharmacoresistant epileptic dogs. Vet J. 2008;176:310‐319. 17468024 10.1016/j.tvjl.2007.03.002

[18] Platt SR , Adams V , Garosi LS , et al. Treatment with gabapentin of 11 dogs with refractory idiopathic epilepsy. Vet Rec. 2006;159:881‐884. 17189599

[19] Dewey CW , Cerda‐Gonzalez S , Levine JM , et al. Pregabalin as an adjunct to phenobarbital, potassium bromide, or a combination of phenobarbital and potassium bromide for treatment of dogs with suspected idiopathic epilepsy. J Am Vet Med Assoc. 2009;235:1442‐1449. 20001779 10.2460/javma.235.12.1442

[20] Dewey CW , Guiliano R , Boothe DM , et al. Zonisamide therapy for refractory idiopathic epilepsy in dogs. J Am Anim Hosp Assoc. 2004;40(4):285‐291. 15238558 10.5326/0400285

[21] von Klopmann T , Rambeck B , Tipold A . Prospective study of zonisamide therapy for refractory idiopathic epilepsy in dogs. J Small Animal Pract. 2007;48:134‐138. 10.1111/j.1748-5827.2006.00290.x 17355603

[22] Andrew SE . Immune‐mediated canine and feline keratitis. Vet Clin North Am Small Anim Pract. 2008;38:269‐290. 18299007 10.1016/j.cvsm.2007.11.007

[23] Muñana KR , Nettifee‐Osborne JA , Bergman RL Jr , Mealey KL . Association between ABCB1 genotype and seizure outcome in collies with epilepsy. J Vet Intern Med. 2012;26(6):1358‐1364. 22998209 10.1111/j.1939-1676.2012.01006.x

[24] Erlen A , Potschka H , Volk HA , Sauter‐Louis C , O'Neill DG . Seizure occurrence in dogs under primary veterinary care in the UK: prevalence and risk factors. J Vet Intern Med. 2018;32(5):1665‐1676. 30216557 10.1111/jvim.15290 PMC6189390

[25] Steinmetz K , Becker‐Bense S , Strobl R , Grill E , Seelos K , Huppert D . Vestibular paroxysmia: clinical characteristics and long‐term course. J Neurol. 2022;269(12):6237‐6245. 35595969 10.1007/s00415-022-11151-6 PMC9618515

[26] Strupp M , Lopez‐Escamez JA , Kim JS , et al. Vestibular paroxysmia: Diagnostic criteria. J Vestib Res. 2016;26(5–6):409‐415. 28262641 10.3233/VES-160589 PMC9249278

[27] Best C , Gawehn J , Krämer HH , et al. MRI and neurophysiology in vestibular paroxysmia: contradiction and correlation. J Neurol Neurosurg Psychiatry. 2013;84(12):1349‐1356. 24006051 10.1136/jnnp-2013-305513

[28] Chen CC , Lee TY , Lee HH , Kuo YH , Bery AK , Chang TP . Vestibular paroxysmia: long‐term clinical outcome after treatment. Front Neurol. 2022;13:1036214. 36313490 10.3389/fneur.2022.1036214 PMC9614226

[29] Fotedar N , Gajera P , Pyatka N , et al. A descriptive study of eye and head movements in versive seizures. Seizure. 2022;98:44‐50. 35417829 10.1016/j.seizure.2022.04.003

[30] Loddenkemper T , Kotagal P . Lateralizing signs during seizures in focal epilepsy. Epilepsy Behav. 2005;7(1):1‐17. 15975856 10.1016/j.yebeh.2005.04.004

[31] Wyllie E , Lüders H , Morris HH , et al. The lateralizing significance of versive head and eye movements during epileptic seizures. Neurology. 1986;36(5):606‐611. 3703259 10.1212/wnl.36.5.606

[32] Baysal‐Kirac L , Rémi J , Loesch AM , Hartl E , Vollmar C , Noachtar S . Eye movements differ between ictal ipsilateral and contralateral head turning. Epilepsy Res. 2015;114:73‐77. 26088888 10.1016/j.eplepsyres.2015.03.016

[33] DeLahunta A , Glass E , Kent M . de Lahunta's Veterinary Neuroanatomy and Clinical Neurology. 5th ed. Philadelphia, PA: Elsevier; 2021:345‐373.

[34] De Rycke LM , Gielen IM , Van Meervenne SA , et al. Computed tomography and cross‐sectional anatomy of the brain in clinically normal dogs. Am J Vet Res. 2005;66(10):1743‐1756. 16273906 10.2460/ajvr.2005.66.1743

[35] Zarelli M , Schwarz T , Puggioni A , Pinilla M , O'Doherty JV , McAllister H . An optimized protocol for multislice computed tomography of the canine brain. Vet Radiol Ultrasound. 2014;55(4):387‐392. 24506077 10.1111/vru.12144

